# *APOE* E4 is associated with impaired self-declared cognition but not disease risk or age of onset in Nigerians with Parkinson’s disease

**DOI:** 10.1038/s41531-022-00411-x

**Published:** 2022-11-12

**Authors:** Njideka U. Okubadejo, Olaitan Okunoye, Oluwadamilola O. Ojo, Babawale Arabambi, Rufus O. Akinyemi, Godwin O. Osaigbovo, Sani A. Abubakar, Emmanuel U. Iwuozo, Kolawole W. Wahab, Osigwe P. Agabi, Uchechi Agulanna, Frank A. Imarhiagbe, Oladunni V. Abiodun, Charles O. Achoru, Akintunde A. Adebowale, Olaleye Adeniji, John E. Akpekpe, Mohammed W. Ali, Ifeyinwa Ani-Osheku, Ohwotemu Arigbodi, Salisu A. Balarabe, Abiodun H. Bello, Oluchi S. Ekenze, Cyril O. Erameh, Temitope H. Farombi, Michael B. Fawale, Morenikeji A. Komolafe, Paul O. Nwani, Ernest O. Nwazor, Yakub Nyandaiti, Emmanuel E. Obehighe, Yahaya O. Obiabo, Olanike A. Odeniyi, Francis E. Odiase, Francis I. Ojini, Gerald A. Onwuegbuzie, Nosakhare Osemwegie, Olajumoke O. Oshinaike, Folajimi M. Otubogun, Shyngle I. Oyakhire, Funlola T. Taiwo, Uduak E. Williams, Simon Ozomma, Yusuf Zubair, Dena Hernandez, Sara Bandres-Ciga, Cornelis Blauwendraat, Andrew Singleton, Henry Houlden, John Hardy, Mie Rizig

**Affiliations:** 1 Neurology Unit, Department of Medicine, Faculty of Clinical Sciences, College of Medicine, University of Lagos, Idi-Araba, Lagos State Nigeria; 2 Neurology Unit, Department of Medicine, Lagos University Teaching Hospital, Idi-Araba, Lagos State Nigeria; 3 Department of Molecular Neuroscience, University College London Institute of Neurology, Queen Square, London, UK; 4 School of Population and Public Health, The University of British Columbia, Vancouver, BC Canada; 5 Institute for Advanced Medical Research and Training, College of Medicine, University of Ibadan, Ibadan, Oyo State Nigeria; 6 Jos University Teaching Hospital, Jos, Plateau State Nigeria; 7 Department of Medicine, Ahmadu Bello University Teaching Hospital, Zaria, Kaduna State Nigeria; 8 Neurology Unit, Benue State University & Benue State University Teaching Hospital, Makurdi, Benue State Nigeria; 9 Department of Medicine, University of Ilorin & University of Ilorin Teaching Hospital, Ilorin, Kwara State Nigeria; 10 University of Benin & University of Benin Teaching Hospital, Benin City, Edo State Nigeria; 11 General Hospital, Isolo, Lagos State Nigeria; 12 Neurology Unit, Department of Medicine, Obafemi Awolowo University & Obafemi Awolowo University Teaching Hospitals Complex, Ile-Ife, Osun State Nigeria; 13 Federal Medical Center, Abeokuta, Ogun State Nigeria; 14 Federal Medical Center, Asaba, Delta State Nigeria; 15 Federal Teaching Hospital, Gombe, Gombe State Nigeria; 16 Asokoro District Hospital, Asokoro, Abuja, Federal Capital Territory Nigeria; 17 Department of Internal Medicine, Delta State University Teaching Hospital, Oghara, Delta State Nigeria; 18 Department of Medicine, College of Health Sciences, Usmanu Danfodiyo University & Usmanu Danfodiyo University Teaching Hospital, Sokoto, Sokoto State Nigeria; 19 Department of Medicine, University of Ilorin Teaching Hospital, Ilorin, Kwara State Nigeria; 20 Neurology Unit, Department of Medicine, Faculty of Medical Sciences, University of Nigeria & University of Nigeria Teaching Hospital, Ituku Ozalla, Enugu State Nigeria; 21 Irrua Specialist Hospital, Irrua, Edo State Nigeria; 22 Chief Tony Anenih Geriatrics Center, University College Hospital, Ibadan, Oyo State Nigeria; 23 Nnamdi Azikiwe University Teaching Hospital, Nnewi, Anambra State Nigeria; 24 Department of Medicine, Madonna University College of Medical Sciences, Elele, Rivers State & Federal Medical Center, Owerri, Imo State Nigeria; 25 University of Maiduguri & University of Maiduguri Teaching Hospital, Maiduguri, Borno State Nigeria; 26 Federal Medical Center, Keffi, Nassarawa State Nigeria; 27 Department of Internal Medicine, Delta State University & Delta State University Teaching Hospital, Oghara, Delta State Nigeria; 28 General Hospital, Lagos Island, Lagos, Lagos State Nigeria; 29 University of Abuja & University of Abuja Teaching Hospital, Gwagwalada, Abuja, Federal Capital Territory Nigeria; 30 University of Port Harcourt Teaching Hospital, Port Harcourt, Rivers State Nigeria; 31 Neurology Unit, Department of Medicine, Faculty of Clinical Sciences, Lagos State University College of Medicine, Ikeja, Lagos State Nigeria; 32 Federal Medical Center, Ebute-Metta, Lagos State Nigeria; 33 Department of Internal Medicine, National Hospital, Abuja, Federal Capital Territory Nigeria; 34 Department of Medicine, University College Hospital, Ibadan, Oyo State Nigeria; 35 Department of Internal Medicine, University of Calabar/University of Calabar Teaching Hospital, Calabar, Cross Rivers State Nigeria; 36 Laboratory of Neurogenetics, National Institute on Aging, National Institutes of Health, Bethesda, MD USA; 37 Center For Alzheimer’s and Related Dementias, NIA, NIH, Bethesda, MD USA; 38 Department of Neuromuscular Disease, UCL Queen Square Institute of Neurology and The National Hospital for Neurology and Neurosurgery, London, UK; 39 Neurogenetics Laboratory, UCL Queen Square Institute of Neurology and The National Hospital for Neurology and Neurosurgery, London, UK

**Keywords:** Cognitive ageing, Parkinson's disease

## Abstract

The relationship between *APOE* polymorphisms and Parkinson’s disease (PD) in black Africans has not been previously investigated. We evaluated the association between *APOE* polymorphic variability and self-declared cognition in 1100 Nigerians with PD and 1097 age-matched healthy controls. Cognition in PD was assessed using the single item cognition question (item 1.1) of the MDS-UPDRS. *APOE* genotype and allele frequencies did not differ between PD and controls (*p* > 0.05). No allelic or genotypic association was observed between *APOE* and age at onset of PD. In PD, *APOE ε*4/*ε*4 conferred a two-fold risk of cognitive impairment compared to one or no *ε*4 (HR: 2.09 (95% CI: 1.13–3.89; *p* = 0.02)), while *APOE ε*2 was associated with modest protection against cognitive impairment (HR: 0.41 (95% CI 0.19–0.99, *p* = 0.02)). Of 773 PD with motor phenotype and *APOE* characterized, tremor-dominant (TD) phenotype predominated significantly in ε2 carriers (87/135, 64.4%) compared to 22.2% in persons with postural instability/gait difficulty (PIGD) (30/135) and 13.3% in indeterminate (ID) (18/135, 13.3%) (*p* = 0.037). Although the frequency of the TD phenotype was highest in homozygous *ε*2 carriers (85.7%), the distribution of motor phenotypes across the six genotypes did not differ significantly (*p* = 0.18). Altogether, our findings support previous studies in other ethnicities, implying a role for *APOE ε*4 and *ε*2 as risk and protective factors, respectively, for cognitive impairment in PD.

## Introduction

The gene encoding apolipoprotein E (*APOE*), located on chromosome 19q13.2, has three commonly described polymorphic alleles (*ε*2, *ε*3, *ε*4) constituting six genotypes in humans (*ε*2/*ε*2, *ε*2/ *ε*3, *ε*2/ *ε*4, *ε*3/ *ε*3, *ε*3/ *ε*4, *ε*4/ *ε*4). Apolipoprotein E plays a vital role in lipid metabolism and has been linked to both vascular and neurodegenerative pathological processes. Allelic and genotypic variability in *APOE* have been extensively explored in Alzheimer’s disease (AD) and other neurodegenerative conditions including Parkinson’s disease (PD)^1,2^. Currently, variability in *APOE* is the strongest known common genetic risk factor for late onset AD, in which the *APOE ε*4 allele increases disease risk and lowers the age at disease onset, whereas the *APOE ε*2 allele confers a protective effect against AD^2–4^. In Northern European Ancestry individuals, homozygous carriers of *APOE ε*4 have up to a 12-fold increased risk for AD compared to non-carriers, whereas there is a weaker but significant effect for incident AD in persons of Yoruba ancestry in Nigeria^1,5–7^.

The relationship between *APOE* polymorphic variability and disease status and age at onset remains unclear for PD, with a significant number of studies yielding inconsistent results. Recent evidence from large genome-wide association studies (GWAS) of Northern European ancestry participants showed no convincing link between *APOE* genotype and PD disease status or age at onset^8^. However, recent meta-analyses combining data from cohorts of varied ethnicities including Europeans, Asians and Latin-Americans showed that the association between *APOE* genotype and PD risk could be ancestry dependent^9^. *APOE ε*4 but not ε2 was shown to be a consistent risk factor for PD in Asian populations but not in Northern European ancestry individuals and Latin-Americans^8,9^. *APOE ε*4 was also shown to be consistently associated with a higher incidence of cognitive decline in patients with PD from Northern European, Asian and Latino backgrounds^9^.

Few studies have examined the role of *APOE* (and specifically *ε*4) in neurodegeneration in Africans^10–12^. To date, the link between *APOE* and PD risk and age at onset or PD related cognitive impairment has not been explored in individuals of black African ancestry within or outside Africa. The interaction with motor phenotype is also unknown. The objective of this study was to examine the role of *APOE* polymorphisms in the genetic susceptibility to PD in Nigerians, and to interrogate possible interactions with age at onset, motor phenotype and cognitive status.

## Results

### Cohort characteristics

The study participants comprised of 1100 Nigerians with PD and 1097 cognitively normal age-matched controls of similar ancestry. Baseline characteristics are shown in Supplementary Table 1 and includes the sex distribution (female: PD—302 (27.5%), controls—382 (34.8%)), mean age at study (years) (PD: 64.6 ± 10.0, controls: 62.7 ± 9.0), mean age at onset of PD (59.6 ± 10.5 years) and median duration of PD (interquartile range (IQR)) 3.0 (3.0) years. A significantly higher proportion of controls in this study were female (*p* = 0.000). Among individuals with PD, mean age at study and mean age at onset did not differ by sex (*p* = 0.15, respectively). Disease duration, median PD stage (Hoehn and Yahr), median MDS UPDRS cognition score and the proportions with abnormal cognition (as defined, i.e. score of 0 or 1 on the MDS UPDRS cognition question) were also similar when compared by sex (*p* > 0.05 for all). In non-parametric (Spearman’s) correlation analysis, MDS UPDRS cognition score was significantly positively correlated with age at study (Rs = 0.145, *p* = 0.000), age at onset of PD (Rs = 0.094, *p* = 0.002) and duration of PD at study (Rs = 0.115, *p* = 0.000).

### *APOE* allelic and genotypic frequency proportions in Nigerians with PD and controls

The allele frequencies of *APOE* in all participants (*n* = 2197) were: ε3 (58.9%), ε4 (29%) and ε2 (12.1%). The genotypic frequencies of *APOE* were as follows for homozygotes (*ε*3/*ε*3 (43.2%), *ε*4/*ε*4 (5.7%), *ε*2/*ε*2 (1.4%)) and heterozygotes (*ε*3/*ε*4 (33%), *ε*2/*ε*3 (12%), *ε*2/*ε*4 (4.7%)), respectively. As shown in Tables 1 and 2 and Supplementary Tables 2 and 3, there was no significant difference in allele (*p* = 0.17) or genotype frequencies (*p* = 0.56) in PD versus controls in this study. No sex differences were observed (*p* > 0.05). Tables 1 and 2 also compare the distribution of *APOE* alleles and genotypes in Nigerian PD patients and controls in this study to data from previous reports describing these frequencies in general populations from different ethnicities^13–15^.

**Table 1:** *APOE* allele distribution in Nigerians with PD and controls in comparison to other normal global and ethnic populations.

Allele *n (%)*									
	All *n* = 2197	PD *n* = 1100	Controls *n* = 1097	Global^13^	Africans^13^	Europeans^14^	Asians^14^	Native Americans^14^	Oceanians^14^
*ε*2	397 (12.1)	204 (12.3)	193 (11.8)	0–38	2.7–11.6	4.4–11.9	0.4–14.0	0.0–1.4	0.0–14.5
*ε*3	1937 (58.9)	959 (57.9)	978 (59.9)	48–94	53.6–85.0	64.0–89.8	62.0–87.0	72.0–91.1	48.6–74.0
*ε4*	955 (29.0)	494 (29.8)	461 (28.2)	3–41	14.3–40.7	6.8–31.0	7.1–24.0	8.9–28.0	26.0–68.0

Denominator for allele frequencies is total allele count (Nigerian cohort present study: all = 3289, PD = 1657, controls = 1632). No significant difference in allele frequencies in PD versus controls in this study. Odds ratios (95% CI) PD versus controls (e2: 0.97 (0.87–1.08), *p* = 0.56; e3: 1.10 (0.97–1.24), *p* = 0.15; e4: 0.94 (0.97–1.03), *p* = 0.17). Global data for alleles from refs. ^13,14^. Global data for alleles (not shown) from ref. ^14^ (Corbo, R. M. et al.) (e2: 0.0–37.5; e3: 8.5–98.0; e4: 0.0–49.0).

**Table 2:** *APOE* genotype distribution in Nigerians with PD and controls in comparison to other normal global and ethnic populations.

Genotype	All participants *n* = 2197	PD *n* = 1100	Controls *n* = 1097	Global^15^	Black^15^	Whites^15^
*ε*2/*ε*2	30 (1.4)	18 (1.6)	12 (1.1)	0.53	1.23	0.50
*ε*2/*ε*3	263 (12.0)	129 (11.7)	134 (12.2)	11.99	13.38	12.71
*ε*2/*ε*4	104 (4.7)	57 (5.2)	47 (4.3)	1.78	3.44	2.21
*ε*3/*ε*3	949 (43.2)	459 (41.7)	490 (44.7)	65.68	47.86	60.16
*ε*3/*ε*4	725 (33.0)	371 (33.7)	354 (32.3)	18.61	30.65	22.43
*ε*4/*ε4*	126 (5.7)	66 (6.0)	60 (5.5)	1.41	3.44	1.99

No significant difference in genotype frequencies in PD versus controls in this study. *P-*value for comparison of genotype frequencies in present study (i.e. PD v. controls) = 0.56. Genotype frequencies for controls as reported by Qin, W. et al.^15^ for global and race categorization as stated in the publication (‘Black’ and ‘White’).

### Association between *APOE* and PD risk, age at onset and cognition status

The Odds ratios (95% CI) for the comparison between PD and controls for allele distribution (Supplementary Table 3) were as follows: ε2: 0.97 (0.87–1.08), *p* = 0.56; ε3: 1.10 (0.97–1.24), *p* = 0.15; ε4: 0.94 (0.97–1.03), *p* = 0.17. Supplementary Table 4 provides data on the association of ε2 and ε4 dose to disease status, demonstrating the lack of association with PD status (p>0.05 for all comparisons).

Supplementary Table 5 and Supplementary Fig. 1 explore the allelic and genotypic relationship of *APOE* to age at onset of PD. Genotypic and allelic genotypes in *APOE* did not influence age at onset of PD, and neither did ε2 or ε4 dose (data not shown; *p* > 0.05 for all iterations).

Table 3 provides data on the relationship of allelic and genotypic variability and ε2 and ε4 dose to cognition status in PD. Homozygosity for ε4 conferred a two-fold increased risk for cognitive impairment in PD (Hazards ratio 2.09 (95% CI 1.13–3.89), *p* = 0.02), whereas the presence of at least one ε2 allele reduced the likelihood of cognitive impairment (HR 0.41 (95% CI 0.19–0.88), *p* = 0.023). None of the 18 PD participants homozygous for ε2 had cognitive impairment.

**Table 3:** Relationship between *APOE* ε4 genotype dose and cognition status among Nigerians with Parkinson’s disease.

	PD with normal cognition *n* = 922	PD with impaired cognition *n* = 121	Hazard ratio (95% CI)	*p*-value^a^
*ε4 dose*				
0	546 (90.1)	60 (9.9)	Reference	
1	322 (86.8)	49 (13.2)	1.38 (0.95–2.03)	0.094
2	54 (81.8)	12 (18.1)	2.09 (1.13–3.89)	0.020
*ε2 dose*				
0	782 (87.3)	114 (12.7)	Reference	
1	122 (94.6)	7 (5.4)	0.41(0.19–0.88)	0.023
2	18 (100.0)	0 (0.0)		

ε4 dose: 0 = ε2/ε2, ε2/ε3, ε3/ε3; 1 = ε3/ε4; 2 = ε4/ε4. ε2 dose: 0 = ε3/ε3, ε3/ε4, ε4/ε4; 1 = ε2/ε3; 2 = ε2/ε2. ε2/ ε4 excluded (regarded as neither risk nor protective factor).

^a^Adjusted for sex and age at onset.

### Association between motor phenotype, cognition and *APOE* variability

773 persons with PD had both APOE genotype and motor phenotype data, and are included in this report. Motor phenotype was tremor dominant (TD) in 438 (56.7%), postural instability/gait difficulty (PIGD) in 244 (31.6%), and indeterminate (ID) in 91 (11.8%). Of these, self-declared abnormal cognition was present in a significantly higher number of PIGD (38/244, 15.6%), compared to TD (37/438, 8.4%) and ID (9/91, 9.9%), *p* = 0.02.

Considering the allele as the exposure, the frequency of motor phenotype varied significantly in ε2 carriers, being highest for TD (87, 64.4%) compared to PIGD (30, 22.2%) and ID (18, 13.3%), *p* = 0.037. There was no significant difference in distribution of motor phenotypes in ε3 and ε4 carriers (*p* = 0.59 and *p* = 0.97, respectively) (Supplementary table 7). The distribution of motor phenotypes per *APOE* genotype is also shown in Supplementary table 7, and did not vary significantly (*p* = 0.18), although the frequency of the TD phenotype was highest overall in homozygous ε2 carriers (12, 85.7%).

## Discussion

This is the largest dataset from individuals of black African ancestry investigated to date describing genetic variability in *APOE* in the context of PD and providing a comparison to ethnically matched otherwise healthy subjects from the same geographical location. In addition, we provide further insight into the distribution of *APOE* in modern populations by adding to the existing data on the frequency of *APOE* alleles and genotypes from the healthy population in Nigeria. *APOE* allelic and genotypic frequencies for our entire cohort had a similar distribution to that reported in populations of African ancestry except for the *ε*4*/ε*4 genotype which was higher than the global average (5.7% in the present study versus 1.41% global) and higher than the average frequency in other black populations (3.44%)^13–15^. As has been described in other publications, the *APOE* ε3 was the most frequent allele, present in 59.9% of the healthy controls in this study (compared to the widely variable global range of 8.5–98.0% derived from populations across all continents)^13^, and within the range of rates reported from modern African populations (48–94%)^13–16^. The frequency of ε4 in our healthy controls (28.2%) is also mid-range of the typical rates (14.3–40.7%) for Africa, in which the highest frequencies are in Central Africans (from 29% in Fon to 40% in Aka pygmies)^13^. The least frequent allele was ε2 (present in 11.8%) and also coincides (though at the higher end of the range) with earlier reports from Africa (2.7–11.6%)^13,17^. The genotype distributions in our healthy controls (ε3/ε3 and ε3/ε4 most common) followed a similar trend with the most recent data for individuals of black ethnicity included in the systematic review by Qin et al.^15^.

Regarding the specific objectives of our study, we found no association between any specific *APOE* allele or genotype and the risk of PD in our population. Our findings corroborate previous observations in other populations, indicating that the distribution of *APOE* alleles (including specifically ε4 carrier rates), ε4 or ε2 allele dosage, and *APOE* genotypes are not significantly different between PD and controls^18,19^. We did not observe the significant over-representation of *APOE* ε2 carriers in PD reported in previous meta-analysis, and our observation is similar with the data presented by William-Gray et al. for the primary cohort of 528 PD and 512 controls, in which the frequency of ε2 was 8.3% for both PD and controls^18–20^.

We found a modest but significant (protective) association of the *APOE* ε2 allele with cognitive status in our cohort, with higher ε2 dosage conferring a lower (but small) risk of self-declared cognitive impairment in individuals with PD. On the other hand, the presence of the ε4 allele conferred a two-fold increased risk of abnormal cognition. These findings align with the postulate of a modest protective effect of ε2 and detrimental impact of ε4 on cognition in PD. Several studies and meta-analyses have demonstrated an over-representation of *APOE* ε4 carriers amongst individuals with PD cognitive impairment and dementia, although others have been equivocal or provided only modest evidence^19,21–24^. Studies including GWAS of neuropathologically confirmed PD have also strengthened the credibility of an association with *APOE* e4 carrier status by demonstrating a significant association with cognitive decline in PD^25,26^. The more appealing explanation of the effect of genetic variability on cognition in PD is that of a cumulative effect conferred by multiple common (often independently low risk) variants (polygenic risk) such as *APOE* ε4. A recent longitudinal genome-wide survival study not only confirmed the notion of an association between *APOE* and cognition in PD, but demonstrated a substantial aggregate association of polygenic progression scores (but not polygenic susceptibility scores) with dementia risk, and proposed diverging genetic architectures of cognitive disease progression and susceptibility^27^. The pathophysiologic basis for *APOE* ε4 and ε2 as risk and protective factors, respectively, for cognitive decline in neurodegenerative disorders have yet to be clearly elucidated although clues to biologically plausible mechanism are emerging^28–30^. *APOE* protein structure (and function) varies per allele due to the single amino acid substitutions that result in greater inherent stability in ε2, which also confers less domain specific interactions that deter neurodegenerative processes (such as amyloid-related damage, synaptic dysfunction, oxidation and inflammation) in contrast to ε4^28,29^. Ultimately, the processes are likely complex, multiple/overlapping and reflect interactions with other genetic and environmental factors and aging.

Our findings regarding the higher frequency of abnormal cognition in the PIGD phenotype corroborate the previously documented association of this motor phenotype with a higher burden of abnormal cognition, greater risk for incident dementia in PD and a faster rate of cognitive decline^31–33^. The motor phenotype distribution in this study is similar to that previously described in our cohort overall (TD 56.5%, PIGD 31.4% and ID 12.1%, respectively)^34^. Although we consider our findings preliminary, the trend towards the less severe tremor-dominant motor phenotype in ε2 carriers may signify a protective effect on motor severity. However, this may also reflect the inherent heterogeneity of the genetics and pathophysiology of PD. Subtypes of PD may have different genetic predictors as demonstrated by Factor and colleagues who found that postural instability with falling (PIF) (proposed as a subtype of PIGD) was inversely associated with *APOE* ε4 (suggesting a protective effect), in contrast to the more severe variant of freezing of gait (FOG)^35^.

We acknowledge the limitations with respect to the measure of cognitive function utilized in this analysis, and understand the inherent challenge with specifically comparing our data with studies that have used more widely recommended and robust measures such as the Montreal Cognitive Assessment (MoCA) and other extensive cognition batteries^36^. The aspiration to provide an albeit exploratory impression of the relationship of *APOE* status to cognition in our population where no prior data exists, coupled with the precedence for the use of the MDS UPDRS single patient-reported cognition question in the absence of more robust assessments provided the rationale for this approach^37^. This single item reportedly is most strongly associated with visuospatial/executive function and delayed recall on the MoCA^37^. As alluded to by Mills and colleagues, the less complicated, more global patient-reported cognitive measure are externally valid and inherently useful. Our study is informative in that with only one question about global cognition, the patient had to sum his or her experiences and give a general response based on the degree of self-assessed severity while avoiding the distraction of more interrogative approaches. In addition, our observations are likely credible because the trend of association between *APOE ε*4 and cognitive impairment occurred despite the similarity in potential clinical confounders such as age at onset, duration of PD and age at study. Although the proportion of PD with cognitive impairment at the median duration of disease in this study using the single MDS UPDRS cognition screen is similar to previous studies employing more robust assessments of cognition, the interpretation of our findings must be cautious. A validation study of the MDS-UPDRS Part 1 for non-motor symptoms compared the single item cognition question to Addenbrooke’s Cognitive Examination (ACE), Scales for Outcome of Parkinson’s disease (SCOPA)-cognitive scale (SCOPACOG) and Frontal Assessment Battery (FAB) found a weak (though positive and statistically significant) correlation for all three cognitive scales (ACE, SCOPA-COG and FAB). The study alluded to the possibility of heterogeneity in cognitive profiles in PD, which makes demonstrating a stronger correlation with a single screening question difficult^38^. We acknowledge that our findings do not imply causality or define a mechanism for the cognitive impairment as no radiological, laboratory or neuropathological studies to exclude other underlying risk factors such as vascular or other co-existing neurodegenerative pathologies was conducted. In addition, the perception of cognitive status represents a combination of participant and/or caregiver’s report, and should be interpreted in this context. Our study is also limited by the draw-back of assessing cognitive profile derived from a static snapshot whereas cognitive decline is an inherently dynamic clinical variable which can occur with disease progression in neurodegenerative disorders such as PD.

In conclusion, our study provides an important dataset describing the association between *APOE* and PD in individuals of black African ancestry, demonstrating a lack of association with disease risk and age at onset and indicating a trend of association of cognitive impairment with *APOE ε*4 and protection by higher doses of *APOE* ε2.

## Methods

Approval of the study protocol was obtained from the institutional health research ethics committees at all participating recruiting sites, the National Health Research Ethics Committee (NHREC) in Nigeria and the ethics committee of the University College London and the National Hospital for Neurology and Neurosurgery, London, United Kingdom. All participants provided written informed consent prior to inclusion in the study.

### Participant recruitment and clinical assessments

A total of 1100 Nigerians with PD and 1097 healthy controls matched by age were included in this cohort study. All controls had an abridged neurological examination and were clinically assessed as cognitively normal based on the absence of any self-declared problems with memory, concentration, orientation or attention. We excluded 83 participants (31 controls and 52 PD participants) with incomplete genotyping data. The excluded participants did not differ from those included based on age at study (*p* = 0.998), male/female ratio (*p* = 0.98) or age at onset of PD (*p* = 0.56). Participants were recruited from an ongoing study being conducted by the Nigeria Parkinson’s Disease Research (NPDR) network in collaboration with the International Parkinson’s Disease Genomics Consortium Africa (IPDGC Africa)^39,40^. The NPDR includes participating sites from tertiary neurology clinics covering all 6 geopolitical regions in Nigeria^39^.

PD diagnosis was based on the United Kingdom Parkinson’s Disease Society Brain Bank (UKPDSBB) criteria^41^. Data available for analysis in this study include baseline demographics (age at study, sex, age at onset of PD, duration of PD (years), disease stage (Hoehn and Yahr) and patient-reported cognitive status. Cognitive status was clinically assessed in individuals with PD. No brain imaging or additional evaluation for aetiology of cognitive impairment was conducted. We used the Movement Disorder Society (MDS) Unified Parkinson’s Disease Rating Scale (UPDRS) (*n* = 988) or the earlier version of the UPDRS (*n* = 112) single item question on cognitive status (Part 1 item 1.1 of the instrument). The response is rated as 0: Normal: No cognitive impairment; (1) Slight: Impairment appreciated by patient or caregiver with no concrete interference with the patient’s ability to carry out normal activities and social interactions; (2) Mild: Clinically evident cognitive dysfunction, but only minimal interference with the patient’s ability to carry out normal activities and social interactions; (3) Moderate: Cognitive deficits interfere with but do not preclude the patient’s ability to carry out normal activities and social interactions and (4) Severe: Cognitive dysfunction precludes the patient’s ability to carry out normal activities and social interactions. The responses for the small sample of 113 with old UPDRS scores were recoded to the most approximate MDS UPDRS score (0→0, 1→1, 2→3 and 3 or 4→4). This convenience was adopted because the previously published formulae for calibration of data allows archival UPDRS Parts II and III data to be accurately transferred to MDS-UPDRS scores but are not accurate for Part I and IV scores^42^. In this study, cognition scores of 0 and 1 were interpreted as PD with normal cognition, whereas scores of 2–4 were regarded as abnormal cognition. Motor phenotype was determined using the method described by Stebbins et al.^34,43^. In summary, the phenotypes are computed using specified MDS UPDRS items for computing tremor score (Part II item 2.10 and Part III items 3.15–3.18 assessing postural, kinetic and rest tremor and rest tremor constancy) and postural instability/gait difficulty (PIGD) score (Part II items 2.12 (walking and balance), 2.13 (freezing), 3.10 (gait), 3.11 (freezing of gait) and 3.12 (postural instability)). The categorization of motor phenotype is based on the MDS-UPDRS TD/PIGD score, which is the mean of the tremor items divided by the mean of the PIGD items. Assignment of phenotype is based on the ratio obtained (TD ≥ 1.15, PIGD ≤ 0.90, indeterminate between 0.90 and 1.15)^43^.

### *APOE* genotyping

DNA was extracted from saliva samples collected using DNA Genotek® saliva kits or from venous whole blood samples using standard protocols. *APOE* genetic variation was determined by genotyping two well established non-synonymous single nucleotide polymorphisms (SNPs): rs429358 and rs7412. The Kompetitive Allele-Specific Polymerase Chain Reaction (PCR) assay (KASP™, LGC Genomics. Herts, UK) was used to genotype both SNPs in 987 participants with PD and 1050 controls^44^. In addition, 113 samples from individuals with PD and 47 controls were genotyped using the Infinium® NeuroChip Consortium Array (Illumina, San Diego, CA, USA)^45,46^. NeuroChip array description and validation of its ability to accurately identify *APOE* genotype calls compared to standard Taqman genotyping is well established^47^. Quality control assessments for the arrays were carried out using PLINK version 1.9 and genotype calls of the rs429358 and rs7412 SNPs were extracted to define the *APOE* alleles^45,46^. SNPs genotypes were assessed for Hardy-Weinberg equilibrium (HWE) using Fisher exact test.

### Data analyses

Cohort characteristics are expressed as counts (%), mean ± SD or medians and compared between groups (PD and controls) using two-tailed X^2^ test for categorical variables (or) analysis of variance (ANOVA) or non-parametric alternative for continuous variables as relevant. Frequency proportions in percent of *APOE* alleles (*ε*2, *ε*3, *ε*4) and genotypes (*ε*2/*ε*2, *ε*2/ *ε*3, *ε*2/ *ε*4, *ε*3/ *ε*3, *ε*3/ *ε*4, *ε*4/ *ε*4) were calculated and compared to published data in subjects from other populations. Logistic regression was used to analyze the association between *APOE* and PD risk, and cognitive performance (in individuals with PD with normal cognition versus PD with impaired cognition). The differences in the frequencies of genotypes and allele between persons with PD and controls, and Hardy-Weinberg equilibrium (HWE) was tested using the Pearson’s Chi-square test. SNP rs429358 was in HWE for both control and cases (*p* = 0.76 and *p* = 0.40, respectively). For SNP rs7412, HWE was preserved in controls (*p* = 0.56) but not in (cases *p* = 0.03) (See Supplementary Fig. 1). Cox proportional hazards regression was used to investigate the influence of *APOE* on age of onset of PD. For all analyses, PD cases and (or) controls were used as the dependent variable and the relevant *APOE* allele, genotype and *ε*4 dose as the independent variables, adjusting where relevant for sex, age at onset or at study in PD and age at recruitment for controls. *APOE ε*4 dose was defined as 0 dose = ε2/ε2, ε2/ ε3 and ε3/ ε3, 1 dose = ε3/ ε4 and 2 doses = ε4/ ε4. Genotype ε2/ε4 was excluded from the analysis because ε2 is considered protective and ε4 is considered a risk variant. Data were analyzed using Stata/MP version 16.0 statistical software (Stata Corporation, College Station, TX: StataCorp LLC).

### Reporting summary

Further information on research design is available in the Nature Research Reporting Summary linked to this article.

## Supplementary information

Supplementary Information

Reporting Summary

## Data Availability

The dataset analyzed during the current study is available from the corresponding author upon reasonable request (e.g. reproducibility of research). Sharing restrictions will be applied to sensitive data to preserve the privacy of participants.

## References

[1] Reitz C, Brayne C, Mayeux R (2011). Epidemiology of Alzheimer disease. Nat. Rev. Neurol..

[2] Lambert JC (2013). Meta-analysis of 74,046 individuals identifies 11 new susceptibility loci for Alzheimer’s disease. Nat. Genet.

[3] Jun, G. R. et al. Protein phosphatase 2A and complement component 4 are linked to the protective effect of APOE ɛ2 for Alzheimer’s disease. *Alzheimers Dement.*10.1002/alz.12607 (2022). 10.1002/alz.12607 PMC9360190 35142023

[4] Bellenguez C (2022). New insights into the genetic etiology of Alzheimer’s disease and related dementias. Nat. Genet.

[5] Farrer LA (1997). Effects of age, sex, and ethnicity on the association between apolipoprotein E genotype and Alzheimer disease. A meta-analysis. APOE and Alzheimer Disease Meta Analysis Consortium. JAMA.

[6] Serrano-Pozo A, Das S, Hyman BT (2021). APOE and Alzheimer’s disease: advances in genetics, pathophysiology, and therapeutic approaches. Lancet Neurol..

[7] Hendrie HC (2014). APOE ε4 and the risk for Alzheimer disease and cognitive decline in African Americans and Yoruba. Int. Psychogeriatr..

[8] Sun R, Yang S, Zheng B, Liu J, Ma X (2019). Apolipoprotein E Polymorphisms and Parkinson disease with or without dementia: a meta-analysis including 6453 participants. J. Geriatr. Psychiatry Neurol..

[9] Li J, Luo J, Liu L, Fu H, Tang L (2018). The genetic association between apolipoprotein E gene polymorphism and Parkinson disease: a meta-analysis of 47 studies. Medicine.

[10] Sawyer K, Sachs-Ericsson N, Preacher KJ, Blazer DG (2009). Racial differences in the influence of the APOE epsilon 4 allele on cognitive decline in a sample of community-dwelling older adults. Gerontology.

[11] Knopman, D. S., Mosley, T. H., Catellier, D. J., Coker, L. H. & Atherosclerosis Risk in Communities Study Brain MRI Study. Fourteen-year longitudinal study of vascular risk factors, APOE genotype, and cognition: the ARIC MRI Study. *Alzheimers Dement*. **5**, 207–214 (2009). 10.1016/j.jalz.2009.01.027 19362884

[12] Akinyemi RO (2022). Dementia in Africa: current evidence, knowledge gaps, and future directions. Alzheimers Dement.

[13] Abondio P (2019). The genetic variability of *APOE* in different human populations and its implications for longevity. Genes (Basel).

[14] Corbo RM, Scacchi R (1999). Apolipoprotein E (APOE) allele distribution in the world. Is APOE*4 a ‘thrifty’ allele?. Ann. Hum. Genet.

[15] Qin W (2021). Race-Related Association between APOE genotype and Alzheimer’s disease: a systematic review and meta-analysis. J. Alzheimers Dis..

[16] Eisenberg DT, Kuzawa CW, Hayes MG (2010). Worldwide allele frequencies of the human apolipoprotein E gene: climate, local adaptations, and evolutionary history. Am. J. Phys. Anthropol..

[17] Kamboh MI, Sepehrnia B, Ferrell RE (1989). Genetic studies of human apolipoproteins. VI. Common polymorphism of apolipoprotein E in blacks. Dis. Markers.

[18] Federoff M, Jimenez-Rolando B, Nalls MA, Singleton AB (2012). A large study reveals no association between APOE and Parkinson’s disease. Neurobiol. Dis..

[19] Williams-Gray CH (2009). Apolipoprotein E genotype as a risk factor for susceptibility to and dementia in Parkinson’s disease. J. Neurol..

[20] Huang X, Chen PC, Poole C (2004). APOE-ε2 allele associated with higher prevalence of sporadic Parkinson disease. Neurology.

[21] Gonzalez-Latapi P, Bayram E, Litvan I, Marras C (2021). Cognitive impairment in Parkinson’s disease: epidemiology, clinical profile, protective and risk factors. Behav. Sci. (Basel).

[22] D’Souza T, Rajkumar AP (2020). Systematic review of genetic variants associated with cognitive impairment and depressive symptoms in Parkinson’s disease. Acta Neuropsychiatr..

[23] Mata IF (2017). Large-scale exploratory genetic analysis of cognitive impairment in Parkinson’s disease. Neurobiol. Aging.

[24] Mata IF (2014). APOE, MAPT, and SNCA genes and cognitive performance in Parkinson disease. JAMA Neurol..

[25] Tan MMX (2021). Genome-wide association studies of cognitive and motor progression in Parkinson’s disease. Mov. Disord..

[26] Tunold JA (2021). APOE and MAPT are associated with dementia in Neuropathologically confirmed Parkinson’s disease. Front. Neurol..

[27] Liu G (2021). Genome-wide survival study identifies a novel synaptic locus and polygenic score for cognitive progression in Parkinson’s disease. Nat. Genet.

[28] Suri S, Heise V, Trachtenberg AJ, Mackay CE (2013). The forgotten APOE allele: a review of the evidence and suggested mechanisms for the protective effect of APOE ɛ2. Neurosci. Biobehav Rev..

[29] Szwedo AA (2022). GBA and APOE impact cognitive decline in parkinson’s disease: a 10-year population-based study. Mov. Disord..

[30] Dilliott AA (2021). Association of apolipoprotein E variation with cognitive impairment across multiple neurodegenerative diagnoses. Neurobiol. Aging.

[31] Michels J (2022). Long-term cognitive decline related to the motor phenotype in Parkinson’s disease. J. Parkinsons Dis..

[32] Arie L, Herman T, Shema-Shiratzky S, Giladi N, Hausdorff JM (2017). Do cognition and other non-motor symptoms decline similarly among patients with Parkinson’s disease motor subtypes? Findings from a 5-year prospective study. J. Neurol..

[33] Burn DJ (2006). Motor subtype and cognitive decline in Parkinson’s disease, Parkinson’s disease with dementia, and dementia with Lewy bodies. J. Neurol. Neurosurg. Psychiatry.

[34] Ojo OO (2021). A cross-sectional comprehensive assessment of the profile and burden of non-motor symptoms in relation to motor phenotype in the Nigeria Parkinson Disease Registry cohort. Mov. Disord. Clin. Pract..

[35] Factor SA (2011). Postural instability/gait disturbance in Parkinson’s disease has distinct subtypes: an exploratory analysis. J. Neurol. Neurosurg. Psychiatry.

[36] Skorvanek M (2018). Global scales for cognitive screening in Parkinson’s disease: critique and recommendations. Mov. Disord..

[37] Mills KA (2016). Cognitive impairment in Parkinson’s disease: association between patient-reported and clinically measured outcomes. Parkinsonism Relat. Disord..

[38] Gallagher DA, Goetz CG, Stebbins G, Lees AJ, Schrag A (2012). Validation of the MDS-UPDRS Part I for nonmotor symptoms in Parkinson’s disease. Mov. Disord..

[39] Ojo OO (2020). The Nigeria Parkinson Disease Registry: process, profile, and prospects of a collaborative project. Mov. Disord..

[40] Rizig M (2021). The International Parkinson Disease Genomics Consortium Africa. Lancet Neurol..

[41] Hughes AJ, Daniel SE, Kilford L, Lees AJ (1992). Accuracy of clinical diagnosis of idiopathic Parkinson’s disease: a clinico-pathological study of 100 cases. J. Neurol. Neurosurg. Psychiatry.

[42] Goetz CG, Stebbins GT, Tilley BC (2012). Calibration of Unified Parkinson’s Disease Rating Scale scores to Movement Disorder Society-Unified Parkinson’s Disease Rating Scale cores. Mov. Disord..

[43] Stebbins GT (2013). How to identify tremor dominant and postural instability/gait difficulty groups with the movement disorder society unified Parkinson’s disease rating scale: comparison with the unified Parkinson’s disease rating scale. Mov. Disord..

[44] Zhang L (2019). Apolipoprotein E polymorphisms contribute to statin response in Chinese ASCVD patients with dyslipidemia. Lipids Health Dis..

[45] Sabir MS (2019). Assessment of APOE in atypical parkinsonism syndromes. Neurobiol. Dis..

[46] Hannon E (2020). Genetic risk for Alzheimer’s disease influences neuropathology via multiple biological pathways. Brain Commun..

[47] Blauwendraat C (2017). NeuroChip, an updated version of the NeuroX genotyping platform to rapidly screen for variants associated with neurological diseases. Neurobiol. Aging.

