# Supplementary material for: APOE E4 is associated with impaired self-declared cognition but not disease risk or age of onset in Nigerians with Parkinson’s disease
Source: NPJ Parkinsons Dis. 2022 Nov 12;8:155. doi: 10.1038/s41531-022-00411-x (PMC9653490; doi:10.1038/s41531-022-00411-x)
Supplement: Supplementary file 1 — Supplementary Information [file 41531_2022_411_MOESM1_ESM.pdf]

SUPPLEMENTARY TABLES

Supplementary Table 1. Cohort characteristics of persons with Parkinson's disease and healthy controls

Characteristics	Parkinson's disease			Controls		
	Total	Female	Male	Total	Female	Male
Study participants (n, %)	1100	302 (27.5)	798 (72.5)	1097	382 (34.8)	715 (65.2)
Age at study, (mean ± SD), years	63.9 ± 10.2	64.6 ± 9.9	63.6 ±10.3	63.4 ± 9.2	62.7 ± 9.0	63.8 ±9.3
Age at onset (mean ± SD), years	59.6 ± 10.5	60.3 ±10.6	59.3 ±10.5			
Duration of PD, (mean ± SD), years	4.3 ± 4.0	4.3 ± 4.2	4.3 ± 3.9			
Hoehn and Yahr stage, median (IQR), years	2.0 (1)	2.0 (1)	2.0 (1)			
MDS UPDRS Cognition score (mean ± SD)	0.50 ± 0.81	0.53 ± 0.82	0.49 ± 0.80			
Abnormal cognition (n, %) **	128 (11.6)	38 (12.6)	90 (11.3)			

Footnote: Mean age at study did not differ significantly between PD and controls overall or by sex ($p>0.05$). Significantly higher proportion of controls were female ($p=0.000$). Age at onset of PD ($p=0.15$), duration of PD in months ($p=0.88$), Hoehn and Yahr median stage ($p=0.06$), MDS UPDRS cognition score ($p=0.43$) and frequency of abnormal cognition ($p=0.55$) within the PD group did not differ by sex. MDS UPDRS cognition score p value was based on non-parametric test; median cognition score in both sexes = 0.

Supplementary Table 2. Association between *APOE* genotypes and PD disease status (overall and by sex)

**Genotype**	**PD** ***n*** **(%)**	**Controls** ***n*** **(%)**	**Odds ratio (95% confidence interval)**	***p-value**
*ε*3/*ε*3	459 (48.4)	490 (51.6)	Reference	
*ε*2/*ε*2	18 (60.0)	12 (40.0)	1.79 (0.84-3.83)	0.13
*ε*2/*ε*3	129 (49.1)	134 (50.9)	1.0 (0.75-1.32)	0.99
ε2/*ε4*	57 (54.8)	47 (45.2)	1.18 (0.78-1.80)	0.43
*ε*3/ *ε*4	371 (51.2)	354 (48.8)	1.11 (0.91-1.36)	0.28
*ε*4/ *ε4*	66 (52.4)	60 (47.6)	1.15 (0.79-1.68)	0.47
**Genotype**	**PD (Males)** ***n*** **(%)**	**Controls (Males)** ***n*** **(%)**	**Odds ratio (95% confidence interval)**	***p-value**
*ε*3/*ε*3	322 (50.4)	317 (49.6)	Reference	
*ε*2/*ε*2	12 (57.1)	9 (42.9)	1.49 (0.60-3.68)	0.39
*ε*2/*ε*3	105 (54.1)	89 (45.9)	1.15 (0.82-1.60)	0.42
ε2/*ε4*	46 (55.4)	37 (44.6)	1.19 (0.74-1.91)	0.46
*ε*3/ *ε*4	269 (54.4)	225 (45.6)	1.17 (0.92-1.49)	0.19
*ε*4/ *ε4*	44 (53.7)	38 (46.3)	1.09 (0.68-1.74)	0.73
**Genotype**	**PD (Females)** ***n*** **(%)**	**Controls (Females)** ***n*** **(%)**	**Odds ratio (95% confidence interval)**	***p-value**
*ε*3/*ε*3	137 (44.2)	173 (55.8)	Reference	
*ε*2/*ε*2	6 (66.7)	3 (33.3)	2.74 (0.67-11.3)	0.16
*ε*2/*ε*3	24 (34.8)	45 (65.2)	0.68 (0.39-1.17)	0.16
ε2/*ε4*	11 (52.4)	10 (47.6)	1.26 (0.51-3.09)	0.61
*ε*3/ *ε*4	102 (44.2)	129 (55.8)	1.00 (0.71-1.40)	0.99
*ε*4/ *ε4*	22 (50.0)	22 (50.0)	1.26 (0.67-2.37)	0.48

Footnote: *Adjusted for sex, age-at onset for PD cases and age at study for controls

Supplementary Table 3. Association between *APOE* alleles and PD disease status (overall and by sex)

Allele	PD *n* (%)	Controls *n* (%)	Odds ratio (95% confidence interval)	*p-value
**All**	1,100	1,097		
*ε*3	588 (48.5)	624 (51.5)	Reference	
*ε*2	18 (60.0)	12 (40.0)	1.79 (0.84-3.82)	0.13
ε4	494 (51.7)	461 (48.3)	1.13 (0.95-1.34)	0.18
**Male participants**				
*ε*3	427 (51.3)	406(48.7)	Reference	
*ε*2	12 (57.1)	9 (42.9)	1.44 (0.58-3.55)	0.43
ε4	359 (54.48)	300 (45.5)	1.13 (0.91-1.39)	0.26
**Female participants**				
*ε*3	161 (42.5)	218 (57.5)	Reference	
*ε*2	6 (66.7)	3 (33.3)	2.94 (0.72-12.1)	0.13
ε4	135 (45.6)	161 (54.4)	1.12 (0.83-1.53)	0.46

Footnote: *Adjusted for, sex, age-at onset for PD cases and age at study for controls

Supplementary Table 4. Association between *APOE* ε4 and ε2 dose and PD disease status

	**PD** *n* **(%)**	**Controls** *n* **(%)**	**Odds ratio (95% confidence interval)**	***p-value**
ε4 dose	n = 1,043	n = 1,050		
0	606 (48.8)	636 (51.2)	Reference	
1	371 (51.2)	354 (48.8)	1.10 (0.91-1.33)	0.32
2	66 (52.4)	60 (47.6)	1.14 (0.78-1.65)	0.50
ε2 dose	n = 1,050	n =1,043		
0	896 (49.8)	904 (50.2)	Reference	
1	129 (49.1)	134 (50.9)	0.95 (0.73-1.23)	0.68
2	18 (60.0)	12 (40.0)	1.69 (0.80-3.58)	0.50

Footnote: ε4 dose: 0 = ε2/ε2, ε2/ ε3, ε3/ ε3, 1= ε3/ ε4, 2= ε4/ ε4; ε2 dose: 0 = ε3/ ε3, ε3/ ε4, ε4/ ε4, 1=ε2/ ε3, 2 = ε2/ ε2; ε2/ ε4 excluded (regarded as neither risk nor protective factor) *Adjusted for sex, age at onset for PD cases and age at study for controls.

Supplementary Table 5. Association between *APOE* polymorphism and Parkinson's disease age of onset (overall and by sex).

Age at onset of PD	ε2	ε3	ε4	*p-value
**All**	**n=18 (%)**	**n=588 (%)**	**n=494 (%)**	
<45	2 (2.1)	51 (53.1)	43 (44.8)	0.52
45-54	3 (1.3)	112 (47.9)	119 (50.9)	
55-64	8 (1.9)	226 (55.3)	175 (42.8)	
65-74	3 (1.0)	159 (54.8)	128 (44.1)	
75-84	2 (3.3)	32 (52.5)	27 (44.3)	
≥85	0 (0.0)	8 (80.0)	2 (20.0)	
**Male**	**n=12 (%)**	**n=427 (%)**	**n=359 (%)**	
<45	2 (2.7)	42 (56.8)	30 (40.5)	0.47
45-54	2 (1.1)	80 (45.5)	94 (53.4)	
55-64	5 (1.68)	167 (56.2)	125 (42.1)	
65-74	2 (1.0)	108 (53.7)	91 (45.3)	
75-84	1 (2.4)	24 (57.1)	17 (40.5)	
≥85	0 (0.0)	6 (75.0)	2 (25.0)	
**Female**	**n=6**	**n=161**	**n=135**	
<45	0 (0.0)	9 (40.9)	13 (59.1)	0.75
45-54	1 (1.72)	32 (55.2)	25 (43.1)	
55-64	3 (2.7)	59 (52.7)	50 (44.6)	
65-74	1 (1.1)	51 (57.3)	37 (41.6)	
75-84	1 (5.3)	8 (42.1)	10 (52.6)	
≥85	0 (0.0)	2 (100.0)	0 (0.0)	

*chi-square test

Supplementary Table 6. Association between *APOE* (genotype, allele, ε2 dose) and self-declared cognitive status in Parkinson's disease

	PD with normal cognition n=972 (%)	PD with impaired cognition n=128 (%)	Hazard ratio (95% CI)	*p-value
**Genotype**				
ε3/ε3 (n=459)	406 (88.5)	53 (11.5)	Ref	
ε2/ε2 (n=18)	18 (100)	0 (0.0)	-	
ε2/ε3 (n=129)	122 (94.6)	7 (5.4)	0.47 (0.21-1.04)	0.06
ε2/ε4 (n=57)	50 (87.7)	7 (12.3)	0.86 (0.39-1.91)	0.71
ε3/ε4 (n=371)	322 (86.8)	49 (13.2)	1.19 (0.80-1.76)	0.39
ε4/ε4 (n=66)	54 (81.8)	12 (18.2)	1.81 (0.97-3.40)	0.62
**Allele**				
ε2 (n=18)	18 (100.0)	0 (0.0)	Chitest; p=0.057	
ε3 (n=588)	528 (89.8)	60 (10.2)		
ε4 (n=494)	426 (86.2)	68 (13.8)		
**ε2 dose**	**n=922**	**n=121**		
0 (n=896)	782 (87.3)	114 (12.7)	Reference	
1 (n=129)	122 (94.5)	7 (5.4)	0.41(0.19-0.88)	0.023
2 (n=18)	18 (100.0)	0 (0.0)		
**ε4 dose**	n=922	n=121		
0	546 (90.1)	60 (9.9)	Reference	
1	322 (86.8)	49 (13.2)	1.38 (0.95-2.03)	0.094
2	54 (81.8)	12 (18.1)	2.09 (1.13-3.89)	0.020

Supplementary Table 7. Association between *APOE* alleles, genotypes and PD motor phenotype.

Motor phenotype	*APOE* allele					
	**ε2**	**p value**	**ε3**	**p value**	**ε4**	**p value**
	n=135 (%)		n=678 (%)		n=347 (%)	
Postural instability/gait difficulty	30 (22.2)		218 (32.2)		109 (31.4)	
Tremor dominant	87 (64.4)	0.037	382 (56.3)	0.59	198 (57.1)	0.97
Indeterminate	18 (13.3)		78 (11.5)		40 (11.5)	
	***APOE* genotype n = 773**					
	**ε2/ε2**	**ε2/ε3**	**ε2/ε4**	**ε3/ ε3**	**ε3/ ε4**	**ε4/ ε4**
	n=14 (%)	n=86 (%)	n=35 (%)	n=326 (%)	n=266 (%)	n=46 (%)
Postural instability/gait difficulty	1 (7.1)	18 (20.9)	11 (31.4)	116 (35.6)	84 (31.6)	14 (30.4)
Tremor dominant	12 (85.7)	57 (66.3)	18 (51.4)	171 (52.5)	154 (57.9)	26 (56.5)
Indeterminate	1 (7.1)	11 (12.8)	6 (17.1)	39 (12.0)	28 (10.5)	6 (13.0)
**p value**	0.18					

SUPPLEMENTARY FIGURE

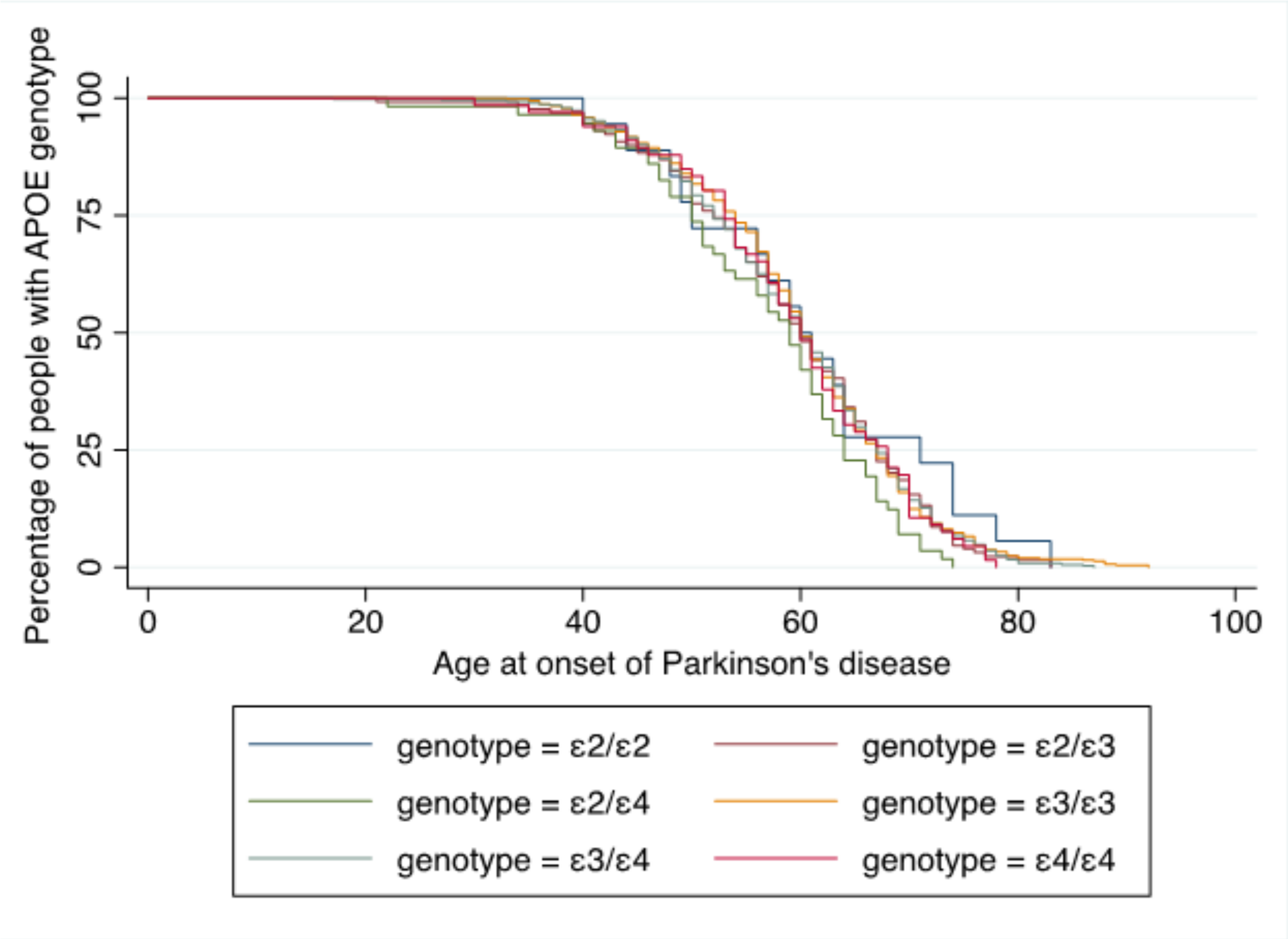

Supplementary Figure 1. Association between APOE genotype and age at onset in persons with Parkinson's disease

Footnote: (p value for comparison = 0.35)
