# Supplementary material for: APOE E4 is associated with impaired self-declared cognition but not disease risk or age of onset in Nigerians with Parkinson’s disease
Source: NPJ Parkinsons Dis. 2022 Nov 12;8:155. doi: 10.1038/s41531-022-00411-x (PMC9653490; doi:10.1038/s41531-022-00411-x)
Supplement: Supplementary file 2 — Reporting Summary [file 41531_2022_411_MOESM2_ESM.pdf]

nature portfolio

Corresponding author(s): PROF. NJIDEKA U. OKUBADEJO

Last updated by author(s): Oct 9, 2022

Reporting Summary

Nature Portfolio wishes to improve the reproducibility of the work that we publish. This form provides structure for consistency and transparency in reporting. For further information on Nature Portfolio policies, see our Editorial Policies and the Editorial Policy Checklist.

Statistics

For all statistical analyses, confirm that the following items are present in the figure legend, table legend, main text, or Methods section.

n/a	Confirmed	
☐	☒	The exact sample size (*n*) for each experimental group/condition, given as a discrete number and unit of measurement
☐	☒	A statement on whether measurements were taken from distinct samples or whether the same sample was measured repeatedly
☐	☒	The statistical test(s) used AND whether they are one- or two-sided<br>*Only common tests should be described solely by name; describe more complex techniques in the Methods section.*
☐	☒	A description of all covariates tested
☐	☒	A description of any assumptions or corrections, such as tests of normality and adjustment for multiple comparisons
☐	☒	A full description of the statistical parameters including central tendency (e.g. means) or other basic estimates (e.g. regression coefficient) AND variation (e.g. standard deviation) or associated estimates of uncertainty (e.g. confidence intervals)
☐	☒	For null hypothesis testing, the test statistic (e.g. *F*, *t*, *r*) with confidence intervals, effect sizes, degrees of freedom and *P* value noted<br>*Give P values as exact values whenever suitable.*
☒	☐	For Bayesian analysis, information on the choice of priors and Markov chain Monte Carlo settings
☒	☐	For hierarchical and complex designs, identification of the appropriate level for tests and full reporting of outcomes
☐	☒	Estimates of effect sizes (e.g. Cohen's *d*, Pearson's *r*), indicating how they were calculated

Our web collection on statistics for biologists contains articles on many of the points above.

Software and code

Policy information about availability of computer code

Data collection	No software used for data collection
Data analysis	Data analyses was conducted using Stata/MP version 16.0 statistical software (StataCorp, College Station, TX)

For manuscripts utilizing custom algorithms or software that are central to the research but not yet described in published literature, software must be made available to editors and reviewers. We strongly encourage code deposition in a community repository (e.g. GitHub). See the Nature Portfolio guidelines for submitting code & software for further information.

Data

Policy information about availability of data

All manuscripts must include a data availability statement. This statement should provide the following information, where applicable:

- Accession codes, unique identifiers, or web links for publicly available datasets
- A description of any restrictions on data availability
- For clinical datasets or third party data, please ensure that the statement adheres to our policy

The datasets generated and/or analyzed during the current study are available from the corresponding author on reasonable request (e.g., reproducibility of research). Sharing restrictions will be applied to sensitive data to preserve the privacy of participants. Other data are available within the article or supplementary materials.

Human research participants

Policy information about studies involving human research participants and Sex and Gender in Research.

Reporting on sex and gender	Sex (biologic attribute) was considered in the study design and was assigned. Disaggregated sex and gender data were not collected in this study. Sex-based analyses was performed for comparison of the Parkinson's disease population with the controls, and for comparison of PD-related clinical characteristics specifically age at onset, median Hoehn & Yahr stage, disease duration, median MDS UPDRS cognition score and proportions with abnormal cognition as well as APOE allele and genotype frequencies
Population characteristics	The co-variate relevant population characteristics for this study are age at study and sex (for cases and controls) and age at study, age at disease onset, sex, disease duration in years, motor phenotype, disease stage (Hoehn & Yahr) for Parkinson's disease cases.
Recruitment	Cases (persons living with Parkinson's disease in Nigeria) were recruited from the Neurology Clinics in participating sites in the Nigeria Parkinson Disease Registry (NPDR). Controls were recruited from the general population and were ethnically and age-matched to the cases. Recruitment in this cohort is still on-going.
Ethics oversight	The National Health Research Ethics Committee of Nigeria, the ethics committee of the University College London and the National Hospital for Neurology and Neurosurgery, London, United Kingdom and the ethics Committees of all participating sites in Nigeria.

Note that full information on the approval of the study protocol must also be provided in the manuscript.

Field-specific reporting

Please select the one below that is the best fit for your research. If you are not sure, read the appropriate sections before making your selection.

☒ Life sciences ☐ Behavioural & social sciences ☐ Ecological, evolutionary & environmental sciences

For a reference copy of the document with all sections, see nature.com/documents/nr-reporting-summary-flat.pdf

Life sciences study design

All studies must disclose on these points even when the disclosure is negative.

Sample size	The overall sample size for the Nigerian cohort in the ongoing study was assigned as 2000 PD and 2000 controls. The current dataset represents ~50% of the ongoing recruitment.
Data exclusions	There were pre-established exclusion criteria e.g. atypical and secondary parkinsonism. In addition, 83 participants (31 controls and 52 PD participants) with incomplete genotyping data were excluded. The excluded participants did not differ from those included based on age at study (p=0.998), male/female ratio (p=0.98), or age at onset of PD (p=0.56).
Replication	Does not apply
Randomization	Randomization not carried out
Blinding	The APOE genotyping was carried out blindly (i.e., the geneticist was blinded to the population status - PD or control.

Reporting for specific materials, systems and methods

We require information from authors about some types of materials, experimental systems and methods used in many studies. Here, indicate whether each material, system or method listed is relevant to your study. If you are not sure if a list item applies to your research, read the appropriate section before selecting a response.

Materials & experimental systems

n/a	Involved in the study
☒	☐ Antibodies
☒	☐ Eukaryotic cell lines
☒	☐ Palaeontology and archaeology
☒	☐ Animals and other organisms
☐	☒ Clinical data
☒	☐ Dual use research of concern

Methods

n/a	Involved in the study
☒	☐ ChIP-seq
☒	☐ Flow cytometry
☒	☐ MRI-based neuroimaging

Clinical data

Policy information about clinical studies

All manuscripts should comply with the ICMJE guidelines for publication of clinical research and a completed CONSORT checklist must be included with all submissions.

Clinical trial registration	This was not a clinical trial
Study protocol	*Note where the full trial protocol can be accessed OR if not available, explain why.*
Data collection	*Describe the settings and locales of data collection, noting the time periods of recruitment and data collection.*
Outcomes	*Describe how you pre-defined primary and secondary outcome measures and how you assessed these measures.*
